# Supplementary material for: Brick Kiln Dataset for Pakistan’s IGP Region Using AI
Source: Sci Data. 2025 May 20;12:830. doi: 10.1038/s41597-025-05148-9 (PMC12092786; doi:10.1038/s41597-025-05148-9)
Supplement: Supplementary file 1 — Brick Kiln Dataset for Pakistan’s IGP Region Using AI [file 41597_2025_5148_MOESM1_ESM.pdf]

Brick Kiln Dataset for Pakistan's IGP Region Using AI— Supplementary Information

April 23, 2025

Introduction

This supplementary document provides additional details related to the methodology employed in this study and the data produced in this study that complement the primary findings presented in the main manuscript. The content herein is designed to offer a comprehensive overview of the data processing steps, mathematical derivations, and extended results referenced in the primary text. This material aims to ensure transparency in the methods used, enhance the reproducibility of the study, and support further exploration by interested researchers.

The structure of this document is as follows: Section I describes the equations and methodology for mapping bounding box centers to geographic coordinates in satellite imagery from Google Maps Static API. Section II outlines the geolocation of brick kilns in the region of interest along with the pollutant emission estimation process from brick kiln operations, complete with equations and emission factor tables.

1 Supplementary Section I

1.1 Equations Mapping Bounding Box Center to Gographical Coordinate

To accurately determine the geographic coordinates corresponding to bounding boxes detected in satellite imagery, we employ a methodical approach involving geospatial interpolation and image analysis. This process begins with acquiring high-resolution satellite images and performing spatial calibration for bounding box localization. Initially, a satellite image for a specified region is obtained. The latitude and longitude change per pixel are computed by measuring the geographic distance between two reference points within the image and the corresponding pixel distance. Mathematically, these quantities are expressed as:

$$\Delta\text{Lat}_{\text{pixel}} = \frac{\Delta\text{Lat}_{\text{geo}}}{W_{\text{image}}} \tag{1}$$

$$\Delta\text{Lon}_{\text{pixel}} = \frac{\Delta\text{Lon}_{\text{geo}}}{W_{\text{image}}} \tag{2}$$

where $\Delta\text{Lat}_{\text{geo}}$ and $\Delta\text{Lon}_{\text{geo}}$ represent the geographic changes in latitude and longitude over the width of the image, and W_{image} denotes the width of the image in pixels. For each bounding box detected within the image, the geographic coordinates of the bounding box center are calculated by determining the pixel offsets from the image center, which is defined as $(W_{\text{image}}/2, H_{\text{image}}/2)$, assuming a square image. The pixel offsets in geographic coordinates are computed using:

$$\Delta\text{Lat}_{\text{bbox}} = (\text{C}_y - \text{C}_{\text{center}}) \times \Delta\text{Lat}_{\text{pixel}} \tag{3}$$

$$\Delta\text{Lon}_{\text{bbox}} = (\text{C}_x - \text{C}_{\text{center}}) \times \Delta\text{Lon}_{\text{pixel}} \tag{4}$$

where C_x and C_y denote the pixel coordinates of the bounding box center, and C_{center} represents the center coordinate of the image.

The final geographic coordinates of the bounding box center are obtained by adding these offsets to the initial geographic coordinates of the image center:

$$\text{Lat}_{\text{bbox}} = \text{Lat}_{\text{center}} + \Delta\text{Lat}_{\text{bbox}} \tag{5}$$

$$\text{Lon}_{\text{bbox}} = \text{Lon}_{\text{center}} + \Delta\text{Lon}_{\text{bbox}} \tag{6}$$

where $\text{Lat}_{\text{center}}$ and $\text{Lon}_{\text{center}}$ are the geographic coordinates of the image center. Since images from the Google Static Maps API are geometrically flattened, the linear relationship between pixel offsets and geographic coordinates remains consistent across the image and the AOI.

2 Supplementary Section II

2.1 Emission Estimation from Brick Kilns

Following the detection of brick kilns, emission estimation is a critical step in assessing their environmental impact. In the study by Guttikunda et al. [1], real-time pollutant monitoring was conducted using portable air quality instruments, which captured near real-time measurements of pollutants such as $PM_{2.5}$ and NO_x. Similarly, Rajarathnam et al. [4] utilized an integrated approach, combining stack emissions measurements from brick kilns with ambient air pollution monitoring to assess overall air quality impacts. Le et al. [2] employed a combination of continuous emissions monitoring systems (CEMS) and field surveys, providing a comprehensive assessment of air pollutants from brick kilns over time.

In contrast, this study adopts a more straightforward, bottom-up approach by estimating emissions using standardized emission factors from the literature [**GAINS2010**, 1, 4]. Although this method lacks the precision and temporal resolution offered by advanced monitoring and modeling techniques, it still allows for the quantification of key pollutants such as NO_x, SO_x, $PM_{2.5}$, and PM_{10}, based on daily production data.

2.1.1 Pollutant Emissions

Assuming coal is the primary fuel used in brick kiln operations, emissions for each pollutant are calculated using established emission factors (in g/kg). These factors represent the quantity of a specific pollutant emitted in grams per kilogram of brick produced. The pollutants considered in this analysis, along with their respective emission factors, daily emissions (kg/day), and seasonal emissions (kg/year), are summarized in Table 1.

Main Formulas

Let E_i represent the emission factor for pollutant i in g/kg. The daily emissions D_i for each pollutant are calculated as:

$$D_i = E_i \times \text{Total Daily Brick Weight (per kiln)}$$

The seasonal emissions S_i for each pollutant are calculated as:

$$S_i = D_i \times 215$$

2.1.2 Seasonal Brick Production

The first step in the emission estimation process involves calculating the daily brick production per kiln, which is essential for quantifying emission levels. According to Mitra and Valette [3], Pakistan produces approximately 45 billion bricks annually. Since the kilns in the target region represent about 65% of the total kilns, the seasonal brick production for this region is adjusted accordingly:

$$\text{Total Seasonal Brick Production (65\%)} = 0.65 \times 45\,\text{billion bricks} = 29.25\,\text{billion bricks}$$

Given there are 11,277 kilns in the study area, the per-day production per kiln is calculated as follows:

$$\text{Daily Production per Kiln} = \frac{29.25\,\text{billion bricks}}{11,277 \times 215} \approx 12,068\,\text{bricks/day}$$

Each brick weighs approximately 3 kg, so the total daily brick weight per kiln is:

$$\text{Daily Brick Weight per Kiln} = 12,068 \times 3 = 36,204\,\text{kg/day per kiln}$$

Due to the kiln operating patterns in Sindh and Punjab, kilns do not operate continuously throughout the year. They typically cease operations during the monsoon season (July to September) and smog season (December to January). Excluding these periods results in approximately 215 operational days per year:

$$\text{Working Days/Year (adjusted)} = 365 - 150 = 215\,\text{days/year}$$

**Pollutant**	**Emission Factor (g/kg)**	**Daily Emissions (kg/day)**	**Seasonal Emissions (kg/year)**
PM_{10}	9.7	351.18	75,503.70
$PM_{2.5}$	6.8	246.19	52,235.35
SO_x	4.6	166.54	35,810.10
NO_x	4.7	170.16	36,581.40

Table 1: Emission factors, daily emissions, and seasonal emissions per kiln, assuming 215 working days per year.

To calculate the daily and seasonal emissions for each pollutant, the total daily brick weight per kiln is multiplied by the respective emission factor. For instance, PM_{10} emissions are estimated at 351.18 kg/day, resulting in seasonal emissions of 75,503.70 kg/year, assuming 215 operational days. This approach is applied across all pollutants, providing an estimate of total pollutant emissions from brick kilns in the region.

3 Supplementary Section III

3.1 Limitations

Random Forest classifier was trained solely on low-resolution RGB channels from Sentinel-2 imagery and not any other spectra. After classification, we applied a post-processing step to remove outliers, identify object centroids through pixel clustering, and used Google Maps Static API to extract high-resolution imagery for YOLO-based object detection. While effective, this process is computationally intensive and time-consuming. Additionally, some Sentinel-2 imagery was not available on Google Earth Engine's API, limiting coverage in certain regions (see Supplementary Figure S2). Incorporating additional Sentinel bands could potentially improve accuracy but would increase the complexity and cost of the pipeline. Additionally, while the study aimed to reduce false positives, the verification of false negatives—undetected brick kilns—was not studied.

The object detection model cannot differentiate between operational and non-operational kilns when the kiln structure remains intact. It is currently limited to distinguishing between kiln types such as Zigzag and FCBK. Future research could integrate satellite data on heat or emissions to improve detection of operational kilns.

Moreover, analysis of $PM_{2.5}$ emissions does not account for daily changes in wind direction, which may affect the exposure levels around kilns. This omission could lead to an incomplete understanding of how emissions disperse and impact surrounding areas.

Additionally, emission factor assumptions, derived from existing literature, may not fully represent local fuel compositions or kiln operations, leading to potential discrepancies in emission estimates. Seasonal operation estimates assume 215 operational days, excluding shutdown during monsoon and smog seasons, but actual kiln activity may vary. Similarly, proximity analysis, limited to a 1 km radius, may not capture

pollutant dispersion influenced by weather and topography, and variations in population vulnerability are not accounted. Kiln type classifications, based on archival satellite imagery, may overlook recent upgrades, potentially affecting emission estimates across kiln types. Limited ground-truth data restricts the scope of validation, and the absence of real-time air quality monitoring near these kilns reduces precision, particularly for pollutants with temporal variability. However, given the scope of this study to present a more simplistic but immediate emission estimates based on the bricks identified. Undoubtedly, there is a need for more comprehensive approach to integrate all the climatic and demographic information.

4 Supplementary Figures

a. b.

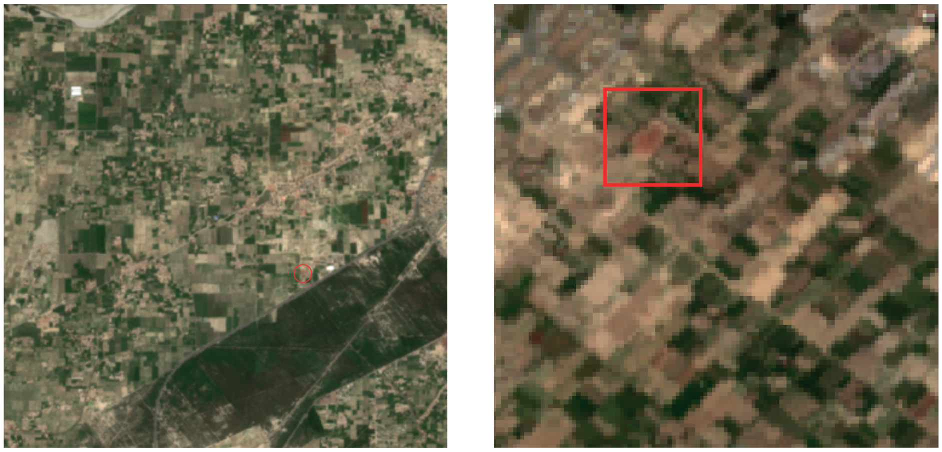

Figure 1: (a) Brick kilns highlighted within a 5x5 km grid. (b) Brick kilns highlighted within a 1x1 km grid.

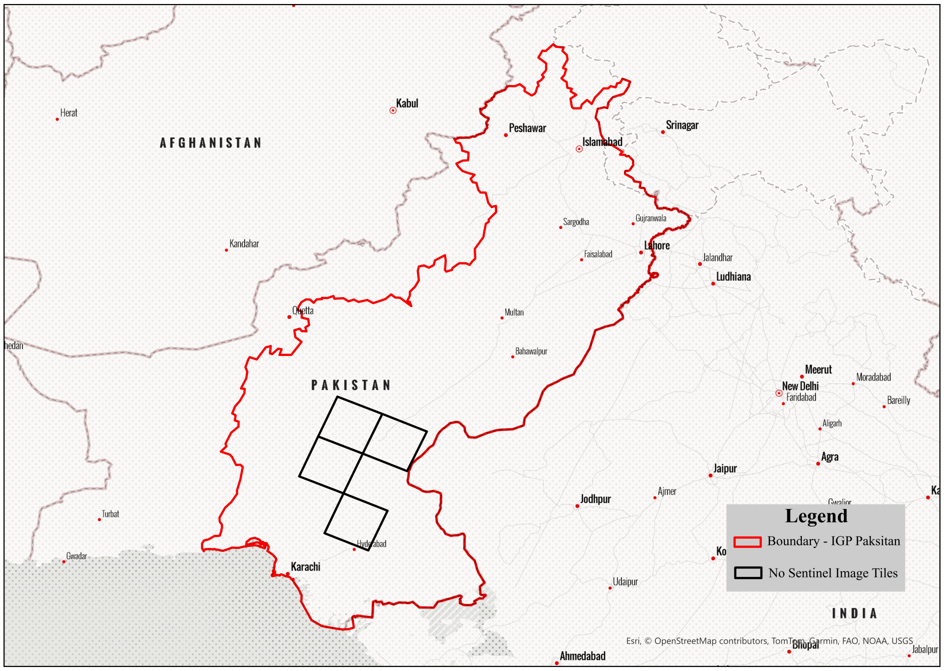

Figure 2: Outlined tiles (black borders) indicate regions where Sentinel-2 imagery was unavailable

5 References

References

[1] Sarath K. Guttikunda, Bilkis A. Begum, and Zia Wadud. “Particulate pollution from brick kiln clusters in the Greater Dhaka region, Bangladesh”. In: *Air Quality, Atmosphere & Health* 6.2 (June 2013), pp. 357–365. ISSN: 1873-9326. DOI: 10.1007/s11869-012-0187-2. URL: https://doi.org/10.1007/s11869-012-0187-2.

[2] Hoang Anh Le and Nguyen Thi Kim Oanh. “Integrated assessment of brick kiln emission impacts on air quality”. In: *Environmental Monitoring and Assessment* 171.1 (Dec. 2010), pp. 381–394. ISSN: 1573-2959. DOI: 10.1007/s10661-009-1285-y. URL: https://doi.org/10.1007/s10661-009-1285-y.

[3] D. Mitra and D. Valette. *Environment, Human Labour, and Animal Welfare: Unveiling the Full Picture of South Asia's Brick Kilns and Building the Blocks for Change*. Geneva, Switzerland: International Labor Office, The Brooke Hospital for Animals, The Donkey Sanctuary, 2017.

[4] Uma Rajarathnam et al. “Assessment of air pollutant emissions from brick kilns”. In: *Atmospheric Environment* 98 (2014), pp. 549–553. ISSN: 1352-2310. DOI: https://doi.org/10.1016/j.atmosenv.2014.08.075. URL: https://www.sciencedirect.com/science/article/pii/S1352231014006888.
